# Sterol metabolism in the filasterean *Capsaspora owczarzaki* has features that resemble both fungi and animals

**DOI:** 10.1098/rsob.160029

**Published:** 2016-07-06

**Authors:** Sebastián R. Najle, María Celeste Molina, Iñaki Ruiz-Trillo, Antonio D. Uttaro

**Affiliations:** 1 Instituto de Biología Molecular y Celular de Rosario (IBR) CONICET and Facultad de Ciencias Bioquímicas y Farmacéuticas, Universidad Nacional de Rosario, Ocampo y Esmeralda s/n, Rosario S2000FHQ, Argentina; 2 Institut de Biologia Evolutiva, CSIC-Universitat Pompeu Fabra, Passeig Marítim de la Barceloneta 37-49, Barcelona 08003, Catalonia, Spain; 3 Departament de Genètica, Universitat de Barcelona, Av. Diagonal, 645, Barcelona 08028, Catalonia, Spain; 4 Institució Catalana de Recerca i Estudis Avançats (ICREA), Passeig Lluís Companys, 23, Barcelona 08010, Catalonia, Spain

**Keywords:** *Capsaspora*, sterol metabolism, transcriptional regulation, opisthokonts, cholesterol, ergosterol

## Abstract

Sterols are essential for several physiological processes in most eukaryotes. Sterols regulate membrane homeostasis and participate in different signalling pathways not only as precursors of steroid hormones and vitamins, but also through its role in the formation of lipid rafts. Two major types of sterols, cholesterol and ergosterol, have been described so far in the opisthokonts, the clade that comprise animals, fungi and their unicellular relatives. Cholesterol predominates in derived bilaterians, whereas ergosterol is what generally defines fungi. We here characterize, by a combination of bioinformatic and biochemical analyses, the sterol metabolism in the filasterean *Capsaspora owczarzaki*, a close unicellular relative of animals that is becoming a model organism. We found that *C. owczarzaki* sterol metabolism combines enzymatic activities that are usually considered either characteristic of fungi or exclusive to metazoans. Moreover, we observe a differential transcriptional regulation of this metabolism across its life cycle. Thus, *C. owczarzaki* alternates between synthesizing 7-dehydrocholesterol de novo, which happens at the cystic stage, and the partial conversion—via a novel pathway—of incorporated cholesterol into ergosterol, the characteristic fungal sterol, in the filopodial and aggregative stages.

## 1. Introduction

Sterols are essential membrane components of most eukaryotic cells, having fundamental structural and signalling functions. As structural components, sterols participate in regulating membrane fluidity and permeability barrier properties [1]. As signalling, sterols act directly as precursors of diverse hormones and bioactive metabolites, including steroids, bile salts and vitamin D in vertebrates [1], ecdysteroids in arthropods [2], dafachronic acids in nematodes [3], brassinosteroids in plants, and antheridiol and oogoniol in fungi [4]. Moreover, together with sphingolipids, sterols are involved in the formation of the lipid rafts, regions of reduced fluidity that selectively incorporate proteins involved in concerted functions such as cell-to-cell recognition, adhesion and communication [5,6]. Thus, sterols play a role in many cellular processes crucial for the development and homeostasis of multicellular organisms, being critical to cell-to-cell communication processes [7–11]. Indeed, alterations in sterol composition and biosynthesis produce several diseases [12].

**Figure 1.**
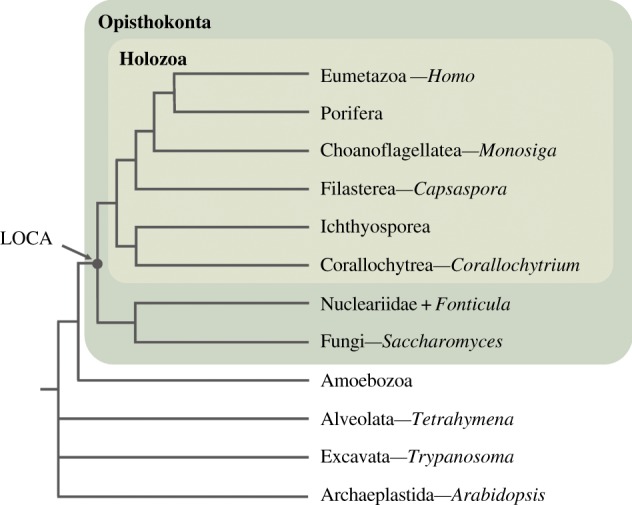
Phylogeny of eukaryotes. Schema of an opisthokont phylogeny (after [19]). Genera mentioned in this study are indicated after the dashes. Dark and light green boxes represent the Opisthokonta and the Holozoa, respectively. LOCA, last opisthokont common ancestor.

Sterols form one of the most diverse families of organic molecules in nature. Interestingly, different eukaryotic groups are usually characterized by having a particular type of sterol, or a reduced number of them, as the major sterol constituent of their membranes. For example, phytosterols (such as sitosterol, C_29_Δ^5^; stigmasterol, C_29_Δ^5,22^; or campesterol, C_28_Δ^5,22^) are the typical sterols found in plants and green algae [13]. On the other hand, ergosterol (ergosta-5,7,22*E*-trien-3β-ol, C_28_Δ^5,7,22^) was historically considered a signature of fungi [14], also used as biomarker for the presence of fungi in the environment [15]. However, by superimposing sterol data available in the literature on a recent phylogeny of fungi, Weete *et al.* [14] suggested that the situation for fungi is more complex. Thus, while ergosterol is indeed the predominant sterol in the most derived fungal clades, cholesterol (cholest-5-en-3β-ol, C_27_Δ^5^) and other Δ^5^ sterols, including 24-methyl and 24-ethyl sterols, are the major sterols in the membranes of the earliest diverging fungal clades, such as Chytridiomycota [14]. Therefore, it seems that the evolution of Holomycota (fungi and their close relatives) was accompanied by a trend from cholesterol-related sterols to ergosterol. Among animals, an opposite situation is observed. Cholesterol predominates in derived bilaterians such as vertebrates, tunicates, annelids, arthropods and molluscs, although other sterols might also be present [13,16,17]. However, early-branching deuterostomes such as echinoderms, and non-bilaterian animals such as cnidarians and poriferans, usually contain an extraordinarily large variety of sterol species, including ergosterol as well as other C24-alkyl sterols [16]. It is worth mentioning the case of sponges, in which more than 70 different sterol species have been characterized (e.g. in the poriferan *Axinella cannabina* [18]; figure 1).

Despite the importance of sterol biogenesis, it remains unclear how the fungal- and animal-like sterol metabolisms emerged. Molecular phylogenies had shown that metazoa and fungi shared a more recent common ancestor than any of them with plants and algae, forming the Opisthokonta clade [20–23]. The opisthokonts also comprise other unicellular organisms, such as choanoflagellates, filastereans, teretosporeans (ichthyosporeans + corallochytreans), nucleariids and *Fonticula alba* (figure 1) [19]. Thus, it is important to analyse sterols in those unicellular lineages to better understand the evolution of sterol metabolism in both fungi and metazoans.

There are three reports so far on sterol metabolism in unicellular holozoans. In one study, it was proposed that ergosterol, like in fungi [24], is the main sterol of the unicellular *Corallochytrium limacisporum,* the sister-group to Ichthyosporea (figure 1). However, the sterol identification was based only on the UV absorption spectra of sterol extracts from this species and compared to those obtained from a wild-type and a Δ*erg3* mutant (deficient in C5-desaturase) strains of the fungus *Neurospora crassa* [24]. In a different study, Kodner *et al.* [25] described the complete sterol profile of the choanoflagellate *Monosiga brevicollis*. The authors showed cholesta-5,7,22-trien-3β-ol (C_27_Δ^5,7,22^) to be the major sterol constituent in *M. brevicollis* membranes and ergosterol as the second most abundant sterol component. In a more recent study, Gold *et al.* [26] explore the sterol composition of the choanoflagellate *Salpingoeca rosetta*, the filasterean *Capsaspora owczarzaki* and the ichthyosporeans *Creolimax fragrantissima* and *Sphaeroforma arctica.* The authors focused on sterol side chain modifications, because they tried to determine whether 24-isopropylcholesterol—a precursor of the sterane 24-isopropylcholestane used as biomarker for sponges [27]—is present in the unicellular relatives of animals. The authors reported *S. rosetta* to have only C_27_ sterols, hence lacking side chain alkylation, and showed ichthyosporeans *C. fragrantissima* and *S. arctica* to possess a wider range of sterols, including C_27_, C_28_ and C_29_ sterols. Moreover, the authors showed cholesterol to be the predominant sterol in *C. owczarzaki*, with trace amounts of ergosterol as well as a few other unidentified minor sterol components [26]. However, there is not yet a systematic analysis of the full repertoire of genes involved in sterol metabolism in unicellular holozoans. Moreover, it is unclear whether and how sterol metabolism is regulated throughout their life cycle.

To fill that gap, we here analysed in detail the sterol metabolism in the filasterean *C. owczarzaki*, a close unicellular relative of animals [23,28]. *Capsaspora owczarzaki* was described as a symbiont of the freshwater snail *Biomphalaria glabrata* from whose haemolymph the unicells were first isolated [29]. *Capsaspora owczarzaki* also offers us the possibility to analyse the dynamics of the sterol metabolism along its life cycle, which includes a filopodial stage, a cystic stage and a multicellular aggregative stage that potentially segregates an extracellular matrix [30]. To understand its sterol metabolism, we performed a bioinformatic survey to identify the gene repertoire of sterol metabolism and analysed their regulation along *C. owczarzaki*'s life cycle. Moreover, we performed biochemical assays to characterize the different sterol synthesis pathways used by this species. We show that sterol metabolism in *C. owczarzaki* is differentially transcriptionally regulated during its life cycle. In the filopodial and aggregative stages, de novo sterol synthesis is repressed, and the cells use cholesterol from the media to produce ergosterol, with sterol neosynthesis restricted to the cystic stage. We also define a potential novel biosynthetic pathway from which *C. owczarzaki* cells produce ergosterol from cholesterol.

## 2. Results

### 2.1. Gene repertoire for sterol synthesis in *Capsaspora owczarzaki*

**Figure 2.**
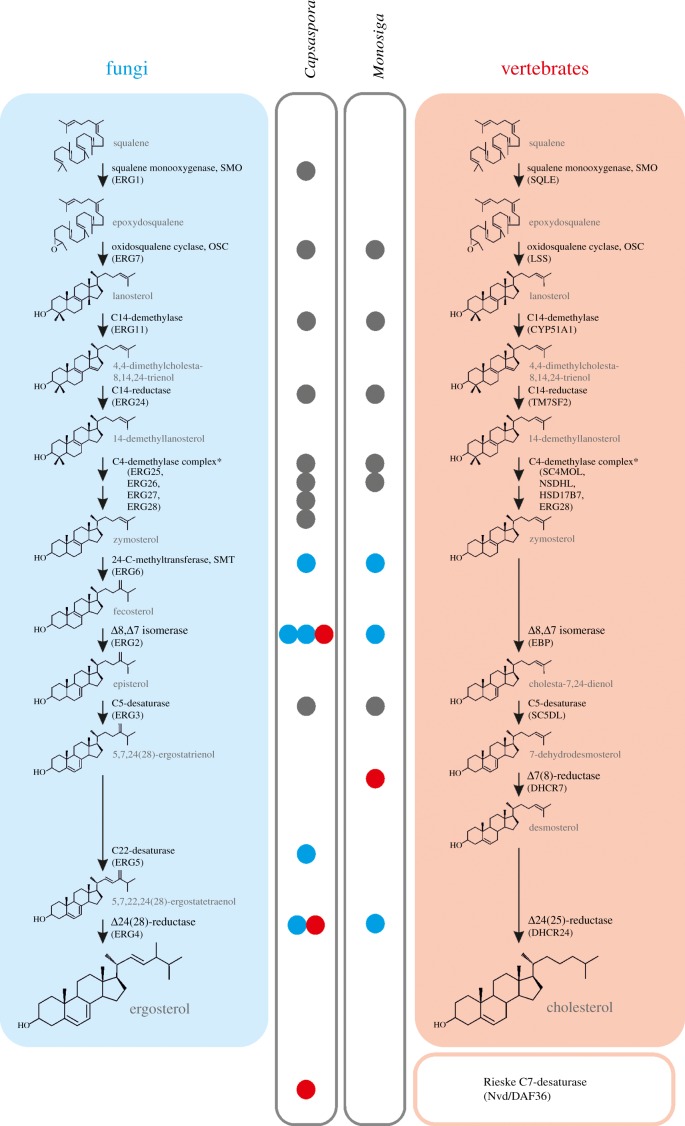
Gene repertoire for sterol biosynthesis in unicellular holozoans. The canonical ergosterol synthesis pathway of fungi and cholesterol synthesis pathway of vertebrates are detailed (blue and red boxes, respectively), with the enzymes involved in each step indicated. Bullets indicate the presence of homologues for the corresponding enzymes in the *C. owczarzaki* and/or *M. brevicollis* genomes, for comparison. Coloured bullets indicate homologues of proteins that are considered exclusive either of fungal (blue) or vertebrate (red) clades. The Rieske sterol C7-desaturase is shown apart as it is not involved, as far as is known, in de novo sterol synthesis.

To have an idea of the gene repertoire for sterol metabolism in *C. owczarzaki*, we mined its genome sequence for genes encoding enzymes involved in the canonical sterol biosynthetic pathways of animals, fungi and plants. We used protein basic local alignment search tool (BLAST [31]) with a set of amino acid sequences of characterized sterol synthesis proteins from *Homo sapiens*, *Saccharomyces cerevisiae* and *Arabidopsis thaliana* as queries. We identified clear orthologues for proteins involved in the ‘canonical’ ergosterol biosynthesis pathway typical of fungi (table 1 and figure 2), as well as other sterol synthesis genes that do not participate in this metabolic route. The presence of orthologues of the first two genes of the pathway, squalene monooxygenase (SMO) and oxidosqualene cyclase (OSC), suggests the capability of *C. owczarzaki* for de novo sterol synthesis. In the canonical sterol pathways, SMO produces squalene epoxide from squalene, and OSC catalyses the conversion of squalene epoxide to the first cyclic product of the pathway (figure 2). The cyclization of epoxidosqualene by OSC might lead to the formation of either lanosterol, typical of vertebrates and fungi, or cycloartenol, as found in plants, algae or *Dictyostelium* [13]. The active site of this highly conserved family of proteins has been largely studied [32], and the production of either lanosterol or cycloartenol by different orthologues can be mainly inferred based on the nature of the amino acid residues at the conserved positions 381, 449 and 453 (numbering relative to the *H. sapiens* OSC) [32]. Fungal and vertebrate lanosterol synthases possess T381, C/Q449 and V453, whereas Y381, H449 and I453 are signatures of cycloartenol synthases [32]. However, there are some exceptions to this rule; amino acid 453 is the only determinant of the enzyme's specificity [13]. The predicted amino acidic sequence of the *C. owczarzaki* OSC shows T381, C449 and V453. This, together with the phylogenetic analysis of squalene and oxidosqualene cyclases (electronic supplementary material, figure S1), strongly suggests that the sterol biosynthetic pathway in this species would begin with the cyclization of epoxidosqualene to lanosterol, which is in agreement with its phylogenetic position within the Opisthokonta, as a close unicellular relative of metazoans [19–21,23,28].

**Table 1.** Sterol biosynthesis genes identified in *C. owczarzaki*.

		similarity to known sequences					
		*Homo sapiens*		*Saccharomyces cerevisiae*		*Arabidopsis thaliana*	
gene description (vertebrate/yeast/plant)	protein ID	score (bits)	ID (%)	score (bits)	ID (%)	score (bits)	ID (%)
squalene monooxygenase, SMO (SQLE/ERG1/SQE)	CAOG_07297	518	55	406	38	307	53
oxidosqualene cyclase, OSC (LSS/ERG7/CAS1)	CAOG_01478	801	54	511	39	629	47
C14-demethylase (CYP51A1/ERG11/CYP51G1)	CAOG_05595	561	52	294	37	322	38
C14-reductase (TM7SF2/ERG24/FK)	CAOG_07273	340	50	310	41	210	37
C4-methyloxidase (SC4MOL/ERG25/SMO1,2)	CAOG_07134	386	60	177	39	204	41
4α-carboxylase 3-dehydrogenase (NSDHL/ERG26/AT3βHSD)	CAOG_05258	401	56	207	36	254	40
hydroxysteroid dehydrogenase 7 (HSD17B7/ERG27/—)	CAOG_04522	234	40	79	23	—	—
C4-demethylase complex D (C14orf1/ERG28/ERG28)	CAOG_04184	96.7	45	76.6	32	48.5	29
24-C-methyltransferase, SMT (—/ERG6/SMT1,2)	CAOG_05252	—	—	316	49	367	52
Δ8,7 isomerase (—/ERG2 /—)	CAOG_04302	—	—	176	51	—	—
	CAOG_00613	—	—	85.5	33	—	—
Δ8,7 isomerase (EBP/—/HYD1)	CAOG_04768	189	52	—	—	77.4	30
C5-desaturase (SC5DL/ERG3/STE1)	CAOG_00853	351	57	256	55	108	34
C22-desaturase (—/ERG5/CYP710A1)	CAOG_00150	—	—	364	39	263	35
Δ24(28)-reductase (—/ERG4/—)	CAOG_01185	—	—	318	40	—	—
Δ24(25)-reductase (DHCR24/—/—)	CAOG_04187	447	44	—	—	401	38
Rieske C7-desaturase^a^ (Nvd,DAF-36/—/—)	CAOG_04775	—	—	—	—	—	—
cyclopropyl isomerase (—/—/CPI1)	CAOG_06065	—	—	—	—	187	38

^a^Orthologues of the Neverland/DAF-36 family of Rieske sterol C7(8)-desaturases are found in vertebrates, except mammals, as well as in some ciliate species [32,33].

Homologues of the enzymes necessary to complete the downstream steps in the pathway were also identified (table 1), with some important differences with respect to choanoflagellates (figure 2). First, contrary to what was reported for *M. brevicollis* [25], a clear orthologue of cytochrome P450 sterol C22-desaturase is found in *C. owczarzaki* (table 1 and figure 2; electronic supplementary material, figure S2). Second, while *M*. *brevicollis* and *Salpingoeca rosetta* each harbour one fungal-like Δ8, Δ7-isomerase (ERG2), *C. owczarzaki* possess two ERG2 homologues as well as a putative protein belonging to the emopamil binding protein (EBP) family, characteristic of vertebrates and plants (table 1 and figure 2) [13]. Regarding the sterol reductase family of proteins (C7-, C14- and C24-reductases), no homologue of sterol C7-reductase is found in *C. owczarzaki*, which is in concordance with an ergosterol pathway. It was reported that *M. brevicollis* does possess an orthologue of C7-reductase [25], a situation that is hard to reconcile with the fact that the three major sterols characterized in this species have a C7(8) double bond [25]. The lack of further biochemical information on sterol metabolism in this species prevents more insights. Other reductases are responsible for the last step in sterol synthesis in both fungi (ERG4) and in vertebrates that follow the Bloch pathway of cholesterol synthesis (DHCR4) [33]. Although both enzymes remove double bonds involving C24 in the lateral chain of sterols, they are non-homologous [13] and carry out reduction reactions on different substrates. Thus, ERG4 is a sterol C24(28)-reductase that converts ergosta-5,7,22,24(28)-tetraen-3β-ol into ergosterol, whereas DHCR24 is a sterol C24(25)-reductase that transforms desmosterol into cholesterol. Interestingly, *C. owczarzaki* has one homologue from each of these C24-reductases (ERG4-like and DHCR24-like; table 1 and figure 2). An orthologue of cyclopropyl sterol isomerase (CPI1) was also identified using the *A. thaliana* CPI1 protein sequence as query (table 1). This observation was also reported by Gold *et al.* [26], who found CPI1 orthologues in several sponge species and in *S. rosetta*. CPI1 enzyme cleaves the cyclopropane ring between carbons C9 and C10 of cycloartenol, and was usually considered to be found in species that are known or predicted to follow the cycloartenol route [13]. Another sterol metabolism enzyme found in *C. owczarzaki* is the Rieske-type sterol C7-desaturase (table 1) [34]. This protein is absent in fungi and highly conserved in animals, except mammals [34,35]. Among unicellular organisms, orthologues of C7-desaturase have been identified in oligohymenophorean ciliates and in the choanoflagellate *S. rosetta*, but not in *M. brevicollis* (figure 2) [34].

### 2.2. Differential expression of sterol biosynthesis genes

**Figure 3.**
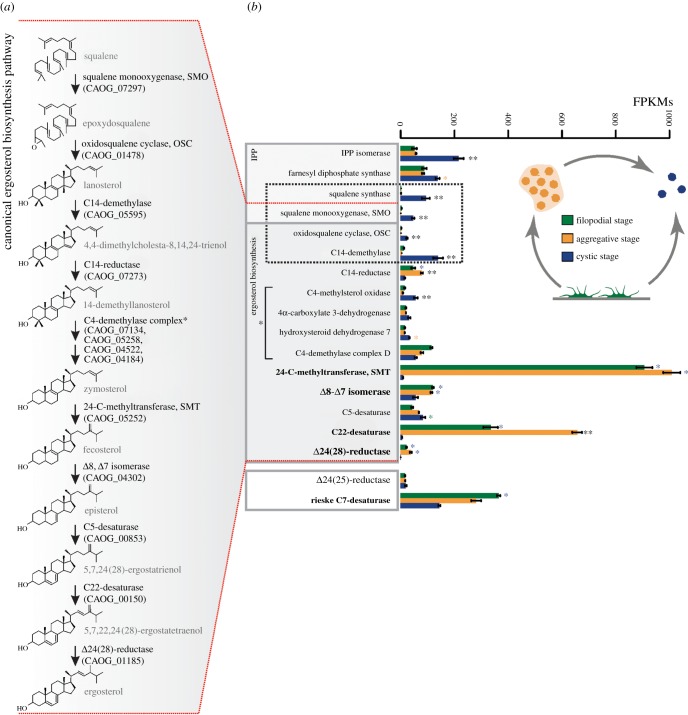
(*a*) Canonical ergosterol synthesis pathway [30]. Identities of the *C. owczarzaki* orthologues of the enzymes involved in each step are indicated in parentheses. (*b*) Expression profile of sterol metabolism genes obtained from RNAseq data [28]. Barplot represents the average of normalized FPKM values for each gene at the three different life cycle stages. Genes coding for the enzymes involved in the first steps of de novo sterol synthesis are highlighted by a dashed box. Enzyme members of the C4-demethylase complex are indicated by a square bracket. Asterisks indicate the gene is significantly differentially expressed in both (two asterisks) or only one (one asterisk) pairwise comparison (aggregative versus filopodial and aggregative versus cystic). Bars show standard error.

To understand the dynamic use of sterol synthesis genes in *C. owczarzaki*, we analysed their expression profiles, including the latest genes for the pre-squalene pathway of isoprenoid synthesis (isopentenyl diphosphate isomerase, farnesyl diphosphate synthase and squalene synthase), from the different *C. owczarzaki* life cycle stages using the data generated in [30] (figure 3). We found that the first four genes in the de novo synthesis pathway (i.e. squalene synthase, SMO, OSC and sterol C14-demethylase; square in figure 3), are exclusively expressed at the cystic stage, being repressed in the filopodial and aggregative stages. Interestingly, a few genes are strongly upregulated in the filopodial and/or aggregative stages with respect to the cystic stage (highlighted in bold in figure 3). These include sterol 24-C-methyltransferase (SMT), C22-desaturase, Δ24(28)-reductase and the Rieske sterol C7-desaturase. Among these, SMT and C22-desaturase are the most highly expressed in both stages, and are also completely repressed in cysts.

### 2.3. Gas chromatography–mass spectrometry profile of *Capsaspora owczarzaki* sterols

**Figure 4.**
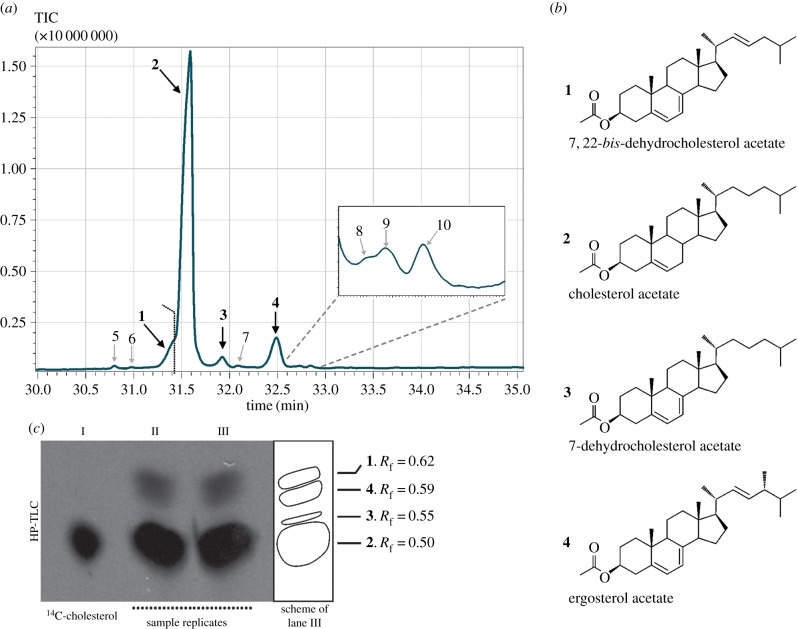
(*a*) Total ion chromatogram (TIC) of acetylated sterols obtained by GC/MS from cultures grown for four days in ATCC 1034 medium. (*b*) The identities and chemical structures of the most abundant compounds are indicated. (*c*) C_18_ HP-TLC autoradiography obtained from *C. owczarzaki* 4-day cultures supplemented with 0.5 µCi 4–^14^C-cholesterol. Lane I: 4–^14^C-cholesterol standard (0.005 µCi); lanes II and III: sterol extracts from two independent cultures. The scheme at the right represents the spots observed in lane III, for better interpretation. Numbering of the sterol species correspond to structures shown in (*b*). *R*_f_, retention factor.

To have a complementary view, we analysed the sterol composition of *C. owczarzaki* membranes obtained from cultures at the filopodial and cystic stages, using gas chromatography–mass spectrometry (GC/MS) as described in Material and methods. Figure 4*a* and electronic supplementary material figure S3*a* show total ion chromatograms (TIC) of the acetylated derivatives from filopodial and cystic stage cells, respectively. No significant differences in sterol profiles were observed between filopodial and cystic stages. Sterol identifications were performed by comparison of the mass spectra obtained with those from commercial standards, published in the literature and/or available in the NIST mass spectral library. Mass spectral fragments of all the sterols identified in the chromatogram are listed in table 2. We identified four dominant compounds (**1–4**, figure 4*a,b*), with cholesterol, **2** (C_27_Δ^5^), being the major constituent, representing 84.17% of the total sterols in the extract. Three other peaks define the bulk of sterol products. In decreasing order of abundance, they were assigned as ergosterol, **4** (C_28_Δ^5,7,22^) (7.76%); 7,22-*bis*-dehydrocholesterol, **1** (C_27_Δ^5,7,22^) (5.04%); and 7-dehydrocholesterol, **3** (C_27_Δ^5,7^) (2.04%). Although the peak corresponding to **1** partially overlaps with that of **2** under our conditions of analysis (figure 4*a*), both compounds were unambiguously identified by differentially subtracting their corresponding mass spectra. Minor peaks in the chromatogram represent 1% of the total of sterols identified and were tentatively assigned to cholesta-5,7,9(11),22-tetraene-3β-ol (peak 5, C_27_Δ^5,7,9(11),22^), 22-dehydrocholesterol (peak 6, C_27_Δ^5,22^), brassicasterol (peak 7, C_28_Δ^5,22^), ergosta-5,7,9(11),22-tetraene-3β-ol (peak 8, C_28_Δ^5,7,9(11),22^), ergosta-5,7,22,24(28)-tetraen-3β-ol (peak 9, C_28_Δ^5,7,22,24(28)^) and campesterol (peak 10, C_28_Δ^5^) (table 2). Structures of all these compounds are detailed in electronic supplementary material, figure S3*b*. No early sterol precursor, namely lanosterol, nor any other 4,4 dimethyl sterol was identified in the profile.

**Table 2.** Mass spectra (70 eV fragments) of acetylated sterols from *C. owczarzaki*.

molecule	TIC peak no.	*m/z* (relative abundance, %)
C_27:3_ Cholesta-5,7,22-triene-3β-ol	1	364 (59), 349 (51), 323 (12), 253 (100), 211 (19), 157 (59), 143 (67), 111 (24), 69 (99), 55 (90)
C_27:1_ Cholest-5-en-3β-ol	2	368 (92), 353 (56), 255 (46), 247 (57), 213 (26), 147 (93), 81 (100), 69 (31), 55 (45)
C_27:2_ Cholesta-5,7-diene-3β-ol	3	366 (59), 351 (71), 253 (100), 211 (22), 143 (90), 69 (79), 55 (64)
C_28:3_ Ergosta-5,7,22-triene-3β-ol	4	378 (47), 363 (36), 253 (89), 211 (15), 199 (18), 157 (50), 143 (54), 69 (100), 55 (64)
C_27:4_ Cholesta-5,7,9(11),22-tetraene-3β-ol	5	422 (1), 362 (47), 347 (29), 251 (76), 235 (46), 209 (27), 69 (100), 55 (74)
C_27:2_ Cholesta-5,22-diene-3β-ol	6	366 (33), 351 (15), 255 (43), 213 (10), 145 (38), 111 (43), 81 (100), 69 (90), 55 (83)
C_28:2_ Ergosta-5,22-diene-3β-ol	7	380 (39), 365 (12), 255 (53), 213 (10), 81 (100), 69 (85), 55 (64)
C_28:4_ Ergosta-5,7,9(11),22-tetraene-3β-ol	8	362 (100), 347 (23), 251 (55), 209 (31), 69 (56), 55 (36)
C_28 :4_ Ergosta-5,7,22,24(28)-tetraene-3β-ol	9	376 (25), 361 (22), 253 (50), 211 (13), 81 (100), 69 (46), 55 (55)
C_28:1_ Ergost-5-en-3β-ol	10	442 (30), 382 (55), 269 (74), 255 (12), 227 (70), 213 (15), 95 (100), 81 (44), 69 (32), 55 (50)

### 2.4. Supplementation of cultures with radioactive precursors

In order to assess the possibility that the cholesterol present in the growth medium is effectively incorporated by the cells and can be further modified for the production of ergosterol, *C. owczarzaki* cultures were supplemented with [^14^C]-cholesterol. After incubation for 4 days, confluent adherent cells were collected, and total lipids were extracted and saponified as described [34]. The non-saponifiable lipid fraction, containing mainly sterols, was analysed by reversed-phase high-performance thin-layer chromatography (HP-TLC) and revealed by autoradiography as detailed in Material and methods. An aliquot of [^14^C]-cholesterol was used as standard, also ensuring the purity of the commercial radiolabelled sterol (lane I in figure 4*c*). Lanes II and III correspond to duplicates from two independent *C. owczarzaki* cultures. Besides the major cholesterol spot (*R*_f_ = 0.50), other spots corresponding to 7-dehydrocholesterol (*R*_f_ = 0.55), ergosterol (*R*_f_ = 0.59) and 7,22-*bis*-dehydrocholesterol (*R*_f_ = 0.62) were identified in the autoradiography (figure 4*c*). To further confirm the identity of the radioactive sterol products, unsaponified lipids from *C. owczarzaki* cultures, supplemented with [^14^C]-cholesterol, were subjected to high-pressure liquid chromatography (HPLC), as described in Materials and methods. We detected radioactive products that eluted at the same retention times as 7,22-*bis*-dehydrocholesterol (20.6 min), ergosterol (25.7 min) and 7-dehydrocholesterol (26.1 min; electronic supplementary material, figure S4).

**Figure 5.**
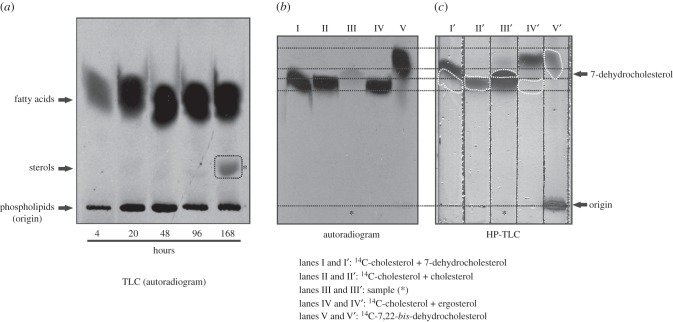
(*a*) Autoradiography from a silica G60 TLC plate of total lipids extracts obtained after growing *C. owczarzaki* cells supplemented with the radioactive precursor 1-^14^C-acetate. All lipid species synthesized de novo are labelled. The picture shows the region of the TLC plate which includes phospholipids, sterols and free fatty acids. Sterols produced de novo can be detected after 168 h (7 days) of incubation with the radioactive precursor (dashed square). The spot (asterisk) was extracted from the TLC plate and analysed on a reverse-phase HP-TLC (C18) system for further characterization (*b,c*). (*b*) Autoradiography of the HP-TLC plate shown in (*c*). The unknown sample (lanes III and III') is compared with standards including both radioactive (*b*) and non-radioactive sterols (*c*) (lanes I and I': ^14^C-cholesterol + 7-dehydrocholesterol; lanes II and II': ^14^C-cholesterol + cholesterol; lanes IV and IV': ^14^C-cholesterol + ergosterol; lanes V and V': ^14^C-7,22-*bis*-dehydrocholesterol + 7,22-*bis*-dehydrocholesterol). Panels (*b,c*) are shown at the same scale, so that they can be superimposed to determine the relative position of each sterol species on the HP-TLC plate (dashed lines and forms). HP-TLC plate was revealed by soaking in ethanol : Cu-phosphoric 1 : 1, and heated at 120°C for 20 min. ^14^C-7,22-*bis*-dehydrocholesterol was obtained from a *Tetrahymena thermophila* culture supplemented with 0.01 µCi ^14^C-cholesterol plus 5 µg ml^−1^ cholesterol, grown for 72 h.

To investigate the capability of sterols neosynthesis in *C. owczarzaki*, 1/100 subcultures were initiated from 4-day cultures (formed by adherent cells under microscopic examination) supplemented with [^14^C]-acetate, and collected at different time points from 4 h up to 7 days post-supplementation. Total lipid extracts were prepared from those cultures and analysed by thin-layer chromatography on SilicaGel G60 plates and revealed by autoradiography (see Material and methods). In this system, lipids can be separated into families based on their polarity, and all sterols species migrate together in a single spot. Figure 5*a* shows that, whereas other lipid classes are produced from as early as 4 h post-incubation with the substrate, sterols were only detected after 7 days of incubation with the radioactive precursor (dashed square in figure 5*a*). This TLC system does not allow the separation of the different sterol species. So, in order to further identify the sterols produced de novo, the spot was scratched from the G60 TLC plate and then analysed by reverse phase HP-TLC using a series of standards as described in Material and methods (figure 5*b,c*). The relative position of each compound could be established by superimposing the autoradiogram with the corresponding HP-TLC plate revealed with Cu : phosphoric reagent (dashed black lines between figure 5*b,c*, and dashed white lines in figure 5*c*). Interestingly, as can be noted in lane III (figure 5*b*), the radioactive sterol species recovered from the cystic cells migrates at the same retention time as 7-dehydrocholesterol standard. Other, non-radioactive sterol species were also present in the sample (lane III' in figure 5*c*), including cholesterol and the triple unsaturated derivatives 7,22-*bis*-dehydrocholesterol and ergosterol. To further characterize de novo synthesized sterols, the unsaponified lipids obtained from cells incubated for 7 days in the presence of the radioactive precursor [^14^C]-acetate, were analysed by HPLC as described in Materials and methods. The profile obtained by absorbance at 210 nm was identical to that in electronic supplementary material, figure S4. However, the radioactivity profile showed a single peak at retention time 26.1 min, indicative of 7-dehydrocholesterol (not shown).

## 3. Discussion

### 3.1. Fungal and metazoan traits are combined in *Capsaspora owczarzaki* sterol pathways

Our data demonstrate that *C. owczarzaki* possesses a peculiar repertoire of sterol metabolism genes, including the complete canonical ergosterol pathway typical of fungi as well as other sterol-metabolism-related genes not involved in this pathway (table 1 and figure 2). This suggests that sterol metabolism in *C. owczarzaki* combines enzymatic activities that are usually considered either characteristic of fungi or exclusive to metazoans. A good example is the coexistence of the two different C24-reductases described above, ERG4-like (fungal) and DHCR24-like (metazoan), that perform similar reactions but potentially act on different substrates (figure 2). Similarly, *C. owczarzaki* possess three putative Δ8,7-isomerases belonging to non-homologous protein families, two homologues of fungal ERG2 and one homologue to the EBP family of proteins, typical of vertebrates and plants. However, phylogenetic analysis suggests that one of the two ERG2 paralogues could be the actual sterol isomerase of *C. owczarzaki*. On the one hand, the other paralogue of this family groups in a different clade with the homologous sigma receptors, members of the opioid receptors family typical of vertebrates, which lacks sterol isomerase activity [36] (electronic supplementary material, figure S2*a*). On the other hand, the EBP homologue, besides conserving the amino acids known to be essential for isomerase activity of EBPs [37], groups to the EBP-like clade in a phylogenetic analysis (electronic supplementary material, figure S2*b,c*). EBP-like proteins lack sterol isomerase activity and their function is still unknown [37,38]. The identity of the real sterol Δ8,Δ7-isomerase in *C. owczarzaki* can be potentially confirmed by heterologous expression of the candidate genes in a Δerg2 yeast strain, but those experiments are beyond the aim of this paper.

Another interesting characteristic of this organism is the Rieske C7-desaturase, with no known orthologues in fungi and highly conserved in animals [34,35]. Among unicellular holozoans, orthologues of C7-desaturase were identified in *C. owczarzaki* and *S. rosetta*, but not in *M. brevicollis* [34] (table 1 and figure 2). Also in contrast to *M. brevicollis*, *C. owczarzaki* possesses an orthologue of the cytochrome P450 sterol C22-desaturase (table 1 and figure 2). This enzyme was postulated to be present in the common ancestor of fungi, animals and plants, and to have been subsequently lost in animals [39]. SMT orthologues are found in both *M. brevicollis* and *C. owczarzaki* genomes (figure 2). SMTs are responsible for the alkylation of the lateral chain of sterols at C24, and are widely conserved in fungi and plants. In animals, orthologues of SMT can be identified in sponges [26] and in the annelid *Capitella teleta*, but are not present in more derived metazoans known to synthesize cholesterol.

Thus, our data on *C. owczarzaki* suggest that the last opisthokont common ancestor had a complex repertoire of genes for sterol metabolism that would allow it to produce a variety of sterol structures. After the divergence of Holozoa and Holomycota, evolutionary canalization leads to derived fungi having ergosterol as the major sterol component of their membranes [14], presumably as an adaptation to live on land [40]. On the other hand, cholesterol predominates in membranes of vertebrates and other bilaterian animals [16].

From a functional point of view, taking into account the particular gene repertoire of *C. owczarzaki*, it is difficult to hypothesize a sterol synthesis pathway in which all these enzymes act in a logical order. For example, the action of Δ8,Δ7-isomerase and Rieske C7-desaturase would redound to sterol products having a double bond in position C7(8). Besides, the presence of both the non-homologous fungal-like and metazoan-like C24-reductases is intriguing. As is described in §3.2, the data hint to a tight regulation of sterol metabolism along the *C. owczarzaki* life cycle.

### 3.2. Dynamic use of sterol metabolism genes in *Capsaspora owczarzaki*

**Figure 6.**
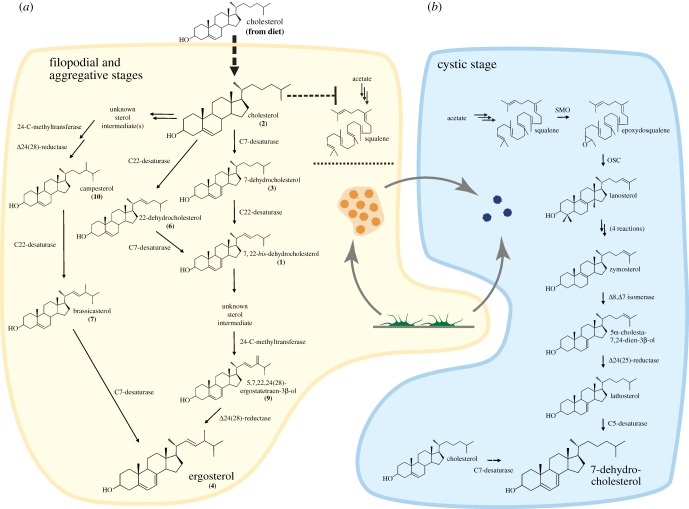
Proposed sterol synthesis pathways for the different life cycle stages in *C. owczarzaki*. (*a*) In both the filopodial and aggregative stages (light orange background), cholesterol (C_27_Δ^5^) is incorporated from the culture medium (dashed arrow) and converted into ergosterol (C_28_Δ^5,7,22^) through desaturations at positions C7(8) and C22(23), and methylation at C24 in the lateral chain. Alternative pathways are depicted, all of them leading to the same main product, ergosterol. De novo synthesis would be inhibited at the transcriptional level (figure 3*b*) as no squalene or cyclic sterol precursor is formed during these stages. Numbers in parentheses accompanying sterol names correspond to peak numbers in figure 4*a*. (*b*) In the cystic stage (light blue background), 7-dehydrocholesterol is produced de novo, and probably also by the Rieske C7(8)-desaturase acting on cholesterol previously incorporated by the cells before encystment, during either the filopodial or aggregative stages.

An important issue is how the different genes for sterol metabolism are orchestrated in these cell stages. The expression profiles of those genes that appear to be differentially transcriptionally regulated along the life cycle (figure 3) [30], together with the biochemical results, lead us to propose a model for sterol metabolism during the life cycle of *C. owczarzaki*. This model should be taken as a working hypothesis. We suggest that the cells alternate between two different metabolic pathways for sterol synthesis during the life cycle (figure 6). Thus, during the filopodial and aggregative stages (yellow background in figure 6), *C. owczarzaki* does not seem to produce sterols de novo. This is supported by the complete repression of the first genes necessary for de novo sterol synthesis (i.e. squalene synthase, SMO, OSC and C14-demetylase) during these stages (dashed square in figure 3*b*). Moreover, by supplementing *C. owczarzaki* cultures with the radioactive precursor [^14^C]-acetate, we further confirmed the absence of neosynthesized sterols during the first 96 h post-incubation with the substrate. At these time points, the cultures are mainly composed of filopodial/adherent and aggregative cells with no cystic cells observable under microscopic examination. On the other hand, after 7 days of incubation with the substrate, when most of the cells in the cultures are in cystic stage, de novo sterol synthesis was evidenced (figure 5*a*).

Our data show that *C. owczarzaki* cystic cells produce 7-dehydrocholesterol de novo (figure 5*b*). Sterol neosynthesis occurring at this stage would start with the production of lanosterol as the first cyclic product. Downstream steps of the pathway would, in principle, be the same as those of the cholesterol synthesis pathway of vertebrates, except for the absence in *C. owczarzaki* of a Δ7-reductase. In vertebrates, this enzyme is responsible of removing the C7(8) double bond from 7-dehydrodesmosterol (cholesta-5,7,24(25)-trien-3β-ol, C_27_Δ^5,7,24(25)^) producing desmosterol [13]. As an inescapable consequence of the lack of Δ7-reductase, the C7(8) double bond generated by Δ8,Δ7-isomerization in a previous step would remain in the final product leading to the production of 7-dehydrocholesterol instead of cholesterol (figure 6*b*).

Another source of 7-dehydrocholesterol in cysts could involve the action of the Rieske C7-desaturase on the cholesterol previously incorporated by the cells before encystment. This possibility is suggested by the relatively high level of expression of this gene in cystic cells (figure 3*b*). Rieske sterol C7-desaturases are highly conserved in animals [34,35], and have a crucial role in development and physiology in ecdysozoans as they catalyse the conversion of diet cholesterol into 7-dehydrocholesterol as the first step in the production of steroid hormones [41,42]. Whether *C. owczarzaki* further modifies 7-dehydrocholesterol for the production of more derived compounds with hormonal functions remains to be determined. At the moment, based on the evidence in favour of the production of 7-dehydrocholesterol de novo in cysts, we think that 7-dehydrocholesterol, even though it is a minor component of membranes, could be important for the physiology of the cystic cells. The presence of both C5(6) and C7(8) double bonds in the B-ring of sterols has been correlated with an increase in resistance to oxidative stress under desiccation in yeasts [40]. It was proposed that these conjugated double bonds allow the stabilization of sterol radicals by resonance, protecting membrane phospholipids against peroxidation [40]. Moreover, the C7(8) double bond is involved in the packing and overall rigidity of the plasma membrane [12,40,43]. Taking into account that *C. owczarzaki* cysts are assumed as a dispersal form [44], we then speculate that the presence of 7-dehydrocholesterol in the membrane is providing cysts with properties of robustness and resistance to dryness, which are useful to face potential tough environmental conditions during dispersion out of the host.

### 3.3. A novel pathway for the conversion of cholesterol into ergosterol

Our data show that the filopodial and aggregative cells take advantage of the cholesterol available in the growth medium, which contains 10% fetal calf serum with a cholesterol concentration of approximately 5 mg ml^−1^. More interestingly, the data strongly suggest that exogenous cholesterol is incorporated by these cells and partially transformed into ergosterol by a previously unknown metabolic pathway (figure 6*a*). More than 80% of incorporated cholesterol remains as such in the cell membranes, whereas the rest is converted into ergosterol (figure 4). Three modifications on the cholesterol moiety are necessary for this conversion: (i) the introduction of a double bond at C7(8) of the B ring, (ii) the introduction of a double bond at position C22(23) of the lateral chain, and (iii) the ramification of the lateral chain by addition of a methyl group at C24 (figure 3*a*). Notably, the three genes coding for the enzymes necessary to carry on the abovementioned modifications (i.e. Rieske sterol C7-desaturase, sterol C22-desaturase and SMT) are strongly upregulated in the active stages (figure 3*b*).

Hence, the production of 7-dehydrocholesterol in *C. owczarzaki* (**3** in figure 4) is explained by the action of the Rieske sterol C7-desaturase on the serum-derived cholesterol (**2** in figure 4). Similarly, the triple unsaturated derivative 7,22-*bis*-dehydrocholesterol (**1**) would be the product of the P450 sterol C22-desaturase acting on **3** (figure 4). As expected, no early sterol precursor, namely lanosterol nor any other 4,4-dimethyl sterol, was identified in the GC/MS profile, further reinforcing the lack of neosynthesis in these stages. Furthermore, we have demonstrated the production of [^14^C]-ergosterol (**4**), as well as the intermediary products [^14^C]-7-dehydrocholesterol (**3**) and [^14^C]-7,22-*bis*-dehydrocholesterol (**1**), by supplementing *C. owczarzaki* cultures with [^14^C]-cholesterol (**2**) (figure 4*c*; electronic supplementary material, figure S4).

In the canonical ergosterol synthesis pathway of yeasts (figure 2 or figure 3*a*), the transfer of the methyl group from *S*-adenosyl-methionine to the sterol Δ24(25) unsaturated chain by SMT produces a sterol with a C24(28) methylene that is reduced in the last step of the pathway by the sterol Δ24(28)-reductase, *ERG4* [45]. Concordantly, we identified a putative tetra-unsaturated sterol in the GC/MS profile that could correspond to ergosta-5,7,22,24(28)-tetraenol (peak 9 in figure 4; table 2 and figure 2*a*; electronic supplementary material, figure S3*b*). Additionally, Δ24(28)-reductase of *C. owczarzaki* showed to be exclusively expressed in filopodial and aggregative stages (figure 3*b*), which is in agreement with our model (figure 6*a*). Substrates known to be recognized by plant and fungal SMTs are preferably Δ24(25)-olefins [46,47]. In our hypothetical pathway, either cholesterol or 7,22-*bis*-dehydrocholesterol should be converted to Δ24(25) derivatives to fulfil this requirement (figure 6*a*). Although there is no enzyme(s) known yet to catalyse this reaction, this does not preclude its existence.

Other minor sterol species detected by GC/MS, whose structures were tentatively assigned to campesterol (peak 10 in figure 4; C_28_Δ^5^), 22-dehydrocholesterol (peak 6 in figure 4; C_27_Δ^5,22^) and brassicasterol (peak 7 in figure 4; C_28_Δ^5,22^) (table 2 and figure 4*a*; electronic supplementary material, figure S3) can be considered as intermediary products of alternative pathways by using the same set of enzymes, leading ultimately to the same end product: ergosterol (figure 6*a*). Another two tetra-unsaturated sterols detected in our analysis were identified as cholesta-5,7,9(11),22-tetraenol (peak 5 in figure 4; C_27_^Δ5,7,9(11),22^) and ergosta-5,7,9(11),22-tetraenol (peak 8 in figure 4; C_28_^Δ5,7,9(11),22^) (table 2 and figure 4*a*; electronic supplementary material, figure S3). Sterols of this series were previously reported in *S. cerevisiae* [48], nematodes [49], poriferans of the class Demospongiae [18,50] and Calcispongiae [51], and in the choanoflagellate *M. brevicollis* [25].

The capacity of *C. owczarzaki* to uptake and modify cholesterol is in consonance with its potential symbiotic lifestyle, as the host snail's haemolymph is a rich source of sterols [52]. Commitment into one route or the other might depend on the opportunity of the cells to incorporate exogenous sterols. We hypothesize that de novo sterol synthesis in filopodial and aggregative cells could be inhibited by the cholesterol incorporated from the medium (figure 4*a*). A similar process occurs in *Trypanosoma brucei*, where de novo synthesis of ergosterol is severely repressed by blood cholesterol in mammalian stages of the parasite [41], although a basal synthesis persists, ensuring the presence of trace amounts of ergosterol as a signalling factor to spark cell proliferation [53,54]. In the ciliate *Tetrahymena thermophila*, the synthesis of the sterol surrogate tetrahymanol is completely repressed after the cells incorporate exogenous sterols [55]. The chance of importing sterols from the environment is also advantageous for these organisms and for *C. owczarzaki*. In principle, only four enzymatic activities are involved in the production of ergosterol from cholesterol (figure 6*a*), compared with the more than 12 enzymatic steps required for ergosterol synthesis from squalene, as occurs in fungi [13]. Interestingly, the two alternative pathways described here are tightly controlled at the transcriptional level (figure 3*b*). Sterol import would be probably impeded in cystic cells, triggering the expression of genes for de novo synthesis of 7-dehydrocholesterol (figure 6*b*). The way the alternative pathways are controlled, and especially the peculiar subset of genes used by filopodial and aggregative cells, as well as their high levels of expression, suggest that ergosterol has an important function in the membranes of these cells. Taking into account the differential expression of the components of the integrin adhesome and signalling partners during these stages [30], proteins known to be associated with lipid rafts [56,57], it will be interesting to investigate the possible involvement of ergosterol in the formation of membrane domains, and hence on the activity of these proteins and their role in aggregative multicellularity.

## 4. Material and methods

### 4.1. Cell culture conditions

*Capsaspora owczarzaki* cells were grown axenically in 25 or 75 cm^2^ flasks containing either 7 or 25 ml of ATCC 1034 medium of the following composition: 1% Bacto peptone, 1% yeast extract, 0.1% ribonucleic acid type VI from Torula yeast, 15 mg l^−1^ folic acid, 1 mg l^−1^ haemin, 10% fetal bovine serum, 2% phosphate buffer (18.1 g l^−1^ KH_2_PO_4_, 25 g l^−1^ Na_2_HPO_4_). Cultivation was carried out in a 23°C incubator. Adherent filopodiated cells were obtained by starting cultures of approximately 5 × 10^4^ cells ml^−1^, after stationary incubation during 3–4 days. Aggregates formation was induced by initiating cultures of approximately 1.5 × 10^4^ cells ml^−1^ and incubated with gentle agitation at 70 r.p.m. for 4–5 days. Floating cystic cells were obtained from 14-day-old stationary cultures, started in the same conditions as the adherent cultures.

### 4.2. Gene expression profiling from RNAseq data

Gene expression analysis was performed using the RNAseq dataset from *C. owczarzaki* obtained by Sebé-Pedrós *et al.* [30], publicly available at NCBI (http://www.ncbi.nlm.nih.gov/biosample/?term=txid595528[Organism:noexp]). Reads were aligned to the reference genome using Tophat [58] with default parameters. Significant differential expression was calculated by performing pairwise comparisons with DESeq [59] (threshold 1 × 10^−5^), EdgeR [60] (threshold 1 × 10^−5^), CuffDiff [58] (threshold 1 × 10^−5^) and NOISeq [61] (threshold 0.8). To be considered as differentially expressed, genes needed to be significant at least in three out of the four methods used.

### 4.3. Identification of sterols by gas chromatography–mass spectrometry

Cells from 4-day-old cultures (adherent) were collected by centrifugation at 3000 *g* for 5 min at 4°C, washed twice with 20 ml of 1X phosphate saline, and the lipids were extracted according to Bligh & Dyer [62]. The organic phase was evaporated to dryness under a N_2_ stream, and the lipids were saponified by incubating at 65°C for 15 min in 1 ml of a mixture of 3 M KOH in methanol and distilled water 1 : 0.9. The reaction was stopped by neutralization with 1 ml of 2 N HCl, and the non-saponifiable lipids were re-extracted twice with 2 ml hexane. The solvent was evaporated under a stream of N_2_, and the residue was resuspended in 50 µl of distilled pyridine. Steryl acetate ester derivatives were obtained by adding 100 µl of acetic anhydride and incubating for 40 min at 80°C. The samples were analysed by running through an SPB-1 column (30 m × 0.25 mm × 0.25 µm; Supelco) in a Shimadzu GC-2010 Plus gas chromatograph. The column was temperature programmed at 5°C min^−1^ from 160 to 320°C and subsequently held for 10 min at 320°C. MS was carried out using a GCMS-QP2010 Plus mass detector, operated at an ionization voltage of 70 eV with a scan range of 20–600 atomic mass units. The retention times and mass spectra of all new peaks obtained were compared with those of standards (Sigma-Aldrich), as well as those available in the literature and in the National Institute of Standards and Technology mass spectral library.

### 4.4. Whole-cell radiolabelling and analysis of sterols by thin-layer chromatography and high-pressure liquid chromatography

*Capsaspora owczarzaki* cultures were initiated from a 1/100 subculture from approximately 5 × 10^6^ cells ml^−1^ initial culture and incubated in ATCC 1034 medium at 23°C with the addition of either 5 µl [4-^14^C]-cholesterol or 10 µl [1-^14^C]-acetic acid (both with specific activity of 55 mCi mmol^−1^; American Radiolabeled Chemicals Inc., St Louis, MO) to a final concentration of 2 or 25 µM, respectively. After the appropriate incubation time, cultures were collected by centrifugation, and lipids were extracted following the methodology of Bligh & Dyer [62] with some modifications. In short, 6 ml of a mixture of chloroform–methanol (1 : 2 by volume) were added to the cell pellet, previously resuspended in 0.8 ml of 1X PBS. After 2 min vortexing, 2 ml of chloroform and 2 ml water were added, vortexed again and centrifuged for 5 min at 2000 *g*. The organic phase was recovered and washed with 4 ml of 2 M KCl by fraction partition, dried under a N_2_ stream, and resuspended in 100 µl chloroform. Sterols from [4-^14^C]-cholesterol labelled cultures were subjected to reverse-phase HP-TLC in Silicagel 60 RP-18 F_254_s plates (Merck Millipore) developed twice with 100% acetone as developing solvent, and revealed by autoradiography using BioMax MR film (Kodak). The retention factors (*R*_f_) for the different compounds were determined with commercial standards from Sigma-Aldrich (cholesterol, 7-dehydrocholesterol and ergosterol) or, in the case of 7,22-*bis*-dehydrocholesterol, by using a sterol extract obtained from culturing *Tetrahymena thermophila* for 72 h in a medium supplemented with cholesterol [34]. Whole lipid extracts from cultures supplemented with [^14^C]-acetate were analysed in Silicagel 60 TLC plates (Merck Millipore) using a mixture of hexane–diethyl ether–acetic acid (40 : 9 : 1 by volume) as developing solvent and revealed by autoradiography as described previously. The sterols fraction was scrapped from the silica, re-extracted twice with 1 ml chloroform and evaporated to dryness under a stream of N_2_. The residue was resuspended in 50 µl chloroform and subjected to reverse-phase HP-TLC, using the same system described above. With the purpose of optimizing the identification of the sterol constituents of the sample, a set of different sterol combinations were used as standards. In all cases, standards included an aliquot of [^14^C]-cholesterol plus a non-radioactive sterol species, namely 7-dehydrocholesterol, cholesterol and ergosterol, with the exception of [^14^C]-7,22-*bis*-dehydrocholesterol obtained from a *T. thermophila* culture incubated in the presence of [^14^C]-cholesterol. The radioactive components of each lane in the HP-TLC plates were evidenced by autoradiography, whereas the non-radioactive sterols were revealed by treating the HP-TLC plate with ethanol : Cu-phosporic reagent 1 : 1 at 120°C for 20 min [63]. Sterols from [4-^14^C]-cholesterol or [1-^14^C]-acetic acid labelled cultures were subjected to HPLC using a Phenomenex Luna C18 column (5 µ, 250 × 4.6 mm). HPLC was developed at 40°C with acetonitrile/water (97 : 3% v/v) isocratically, with a flow rate of 1 ml per min.

## Supplementary Material

Supplementary Table, Figures and Figures Legends (S1–S4)

## Data accessibility

Gene expression analysis was performed using the RNAseq dataset from *C. owczarzaki* obtained by Sebé-Pedrós *et al.* [30], publicly available at NCBI 440 (http://www.ncbi.nlm.nih.gov/biosample/?term=txid595528[Organism:noexp]).

The remaining data supporting this article have been uploaded as part of the electronic supplementary material.

## References

[1] Chang T-Y, Chang CCY, Ohgami N, Yamauchi Y 2006 Cholesterol sensing, trafficking, and esterification. Annu. Rev. Cell Dev. Biol. 22, 129–157. (doi:10.1146/annurev.cellbio.22.010305.104656) 16753029 10.1146/annurev.cellbio.22.010305.104656

[2] Gilbert LI, Rybczynski R, Warren JT 2002 Control and biochemical nature of the ecdysteroidogenic pathway. Annu. Rev. Entomol. 47, 883–916. (doi:10.1146/annurev.ento.47.091201.145302) 11729094 10.1146/annurev.ento.47.091201.145302

[3] Motola DL et al. 2006 Identification of ligands for DAF-12 that govern Dauer formation and reproduction in *C. elegans*. Cell 124, 1209–1223. (doi:10.1016/j.cell.2006.01.037) 16529801 10.1016/j.cell.2006.01.037

[4] Benveniste P 2002 Sterol metabolism. Arabidopsis Book 1, e0004 (doi:10.1199/tab.0004) 22303189 10.1199/tab.0004 PMC3243374

[5] Head BP, Patel HH, Insel PA 2014 Interaction of membrane/lipid rafts with the cytoskeleton: impact on signaling and function: membrane/lipid rafts, mediators of cytoskeletal arrangement and cell signaling. Biochim. Biophys. Acta 1838, 532–545. (doi:10.1016/j.bbamem.2013.07.018) 23899502 10.1016/j.bbamem.2013.07.018 PMC3867519

[6] Lingwood D, Simons K 2010 Lipid rafts as a membrane-organizing principle. Science 327, 46–50. (doi:10.1126/science.1174621) 20044567 10.1126/science.1174621

[7] Resnik N, Sepcic K, Plemenitas A, Windoffer R, Leube R, Veranic P 2011 Desmosome assembly and cell–cell adhesion are membrane raft-dependent processes. J. Biol. Chem. 286, 1499–1507. (doi:10.1074/jbc.M110.189464) 21071449 10.1074/jbc.M110.189464 PMC3020758

[8] Okada Y, Nishikawa J, Semma M, Ichikawa A 2014 Role of lipid raft components and actin cytoskeleton in fibronectin-binding, surface expression, and *de novo* synthesis of integrin subunits in PGE_2_- or 8-Br-cAMP-stimulated mastocytoma P-815 cells. Biochem. Pharmacol. 88, 364–371. (doi:10.1016/j.bcp.2014.01.039) 24518258 10.1016/j.bcp.2014.01.039

[9] Stahley SN, Saito M, Faundez V, Koval M, Mattheyses AL, Kowalczyk AP 2014 Desmosome assembly and disassembly are membrane raft-dependent. PLoS ONE 9, e87809 (doi:10.1371/journal.pone.0087809) 24498201 10.1371/journal.pone.0087809 PMC3907498

[10] Zou J, Yue X-Y, Zheng S-C, Zhang G, Chang H, Liao Y-C, Zhang Y, Xue M-Q, Qi Z 2014 Cholesterol modulates function of connexin 43 gap junction channel via PKC pathway in H9c2 cells. Biochim. Biophys. Acta 1838, 2019–2025. (doi:10.1016/j.bbamem.2014.04.016) 24780378 10.1016/j.bbamem.2014.04.016

[11] Grison MS et al. 2015 Specific membrane lipid composition is important for plasmodesmata function in *Arabidopsis*. Plant Cell 27, 1228–1250. (doi:10.1105/tpc.114.135731) 25818623 10.1105/tpc.114.135731 PMC4558693

[12] Megha BO, London E 2006 Cholesterol precursors stabilize ordinary and ceramide-rich ordered lipid domains (lipid rafts) to different degrees. Implications for the Bloch hypothesis and sterol biosynthesis disorders. J. Biol. Chem. 281, 21 903–21 913. (doi:10.1074/jbc.M600395200) 10.1074/jbc.M600395200 16735517

[13] Desmond E, Gribaldo S 2009 Phylogenomics of sterol synthesis: insights into the origin, evolution, and diversity of a key eukaryotic feature. Genome Biol. Evol. 1, 364–381. (doi:10.1093/gbe/evp036) 20333205 10.1093/gbe/evp036 PMC2817430

[14] Weete JD, Abril M, Blackwell M 2010 Phylogenetic distribution of fungal sterols. PLoS ONE 5, 3–8. (doi:10.1371/journal.pone.0010899) 10.1371/journal.pone.0010899 PMC2878339 20526375

[15] Mille-Lindblom C, von Wachenfeldt E, Tranvik LJ 2004 Ergosterol as a measure of living fungal biomass: persistence in environmental samples after fungal death. J. Microbiol. Methods 59, 253–262. (doi:10.1016/j.mimet.2004.07.010) 15369861 10.1016/j.mimet.2004.07.010

[16] Urich K 1994 Comparative animal biochemistry. Heidelberg, Germany: Springer.

[17] Nelson MM, Phleger CF, Mooney BD, Nichols PD 2000 Lipids of gelatinous Antarctic zooplankton: Cnidaria and Ctenophora. Lipids 35, 551–559. (doi:10.1007/s11745-000-555-5) 10907790 10.1007/s11745-000-555-5

[18] Itoh T, Sica D, Djerassi C 1983 Minor and trace sterols in marine invertebrates. Part 35. Isolation and structure elucidation of seventy-four sterols from the sponge *Axinella cannabina*. J. Chem. Soc. Perkin Trans. 1, 147–153. (doi:10.1039/P19830000147)

[19] Torruella G et al. 2015 Phylogenomics reveals convergent evolution of lifestyles in close relatives of animals and fungi. Curr. Biol. 25, 2404–2410. (doi:10.1016/j.cub.2015.07.053) 26365255 10.1016/j.cub.2015.07.053

[20] Ruiz-Trillo I, Inagaki Y, Davis LA, Sperstad S, Landfald B, Roger AJ 2004 *Capsaspora owczarzaki* is an independent opisthokont lineage. Curr. Biol. 14, R946–R947. (doi:10.1016/j.cub.2004.10.037) 15556849 10.1016/j.cub.2004.10.037

[21] Steenkamp ET, Wright J, Baldauf SL 2006 The protistan origins of animals and fungi. Mol. Biol. Evol. 23, 93–106. (doi:10.1093/molbev/msj011) 16151185 10.1093/molbev/msj011

[22] Cavalier-Smith T, Chao EE-Y 2003 Phylogeny of choanozoa, apusozoa, and other protozoa and early eukaryote megaevolution. J. Mol. Evol. 56, 540–563. (doi:10.1007/s00239-002-2424-z) 12698292 10.1007/s00239-002-2424-z

[23] Ruiz-Trillo I, Roger AJ, Burger G, Gray MW, Lang BF 2008 A phylogenomic investigation into the origin of metazoa. Mol. Biol. Evol. 25, 664–672. (doi:10.1093/molbev/msn006) 18184723 10.1093/molbev/msn006

[24] Sumathi JC, Raghukumar S, Kasbekar DP, Raghukumar C 2006 Molecular evidence of fungal signatures in the marine protist *Corallochytrium limacisporum* and its implications in the evolution of animals and fungi. Protist 157, 363–376. (doi:10.1016/j.protis.2006.05.003) 16899404 10.1016/j.protis.2006.05.003

[25] Kodner RB, Summons RE, Pearson A, King N, Knoll AH 2008 Sterols in a unicellular relative of the metazoans. Proc. Natl Acad. Sci. USA 105, 9897–9902. (doi:10.1073/pnas.0803975105) 18632573 10.1073/pnas.0803975105 PMC2481317

[26] Gold DA, Grabenstatter J, Mendoza AD, Riesgo A, Ruiz-trillo I, Summons RE 2016 Sterol and genomic analyses validate the sponge biomarker hypothesis. Proc. Natl Acad. Sci. USA 113, 2684–2689. (doi:10.1073/pnas.1512614113) 26903629 10.1073/pnas.1512614113 PMC4790988

[27] Love GD et al. 2009 Fossil steroids record the appearance of Demospongiae during the Cryogenian period. Nature 457, 718–721. (doi:10.1038/nature07673) 19194449 10.1038/nature07673

[28] Torruella G, Derelle R, Paps J, Lang BF, Roger AJ, Shalchian-Tabrizi K, Ruiz-Trillo I 2012 Phylogenetic relationships within the Opisthokonta based on phylogenomic analyses of conserved single-copy protein domains. Mol. Biol. Evol. 29, 531–544. (doi:10.1093/molbev/msr185) 21771718 10.1093/molbev/msr185 PMC3350318

[29] Hertel LA, Bayne CJ, Loker ES 2002 The symbiont *Capsaspora owczarzaki*, nov. gen. nov. sp., isolated from three strains of the pulmonate snail *Biomphalaria glabrata* is related to members of the Mesomycetozoea. Int. J. Parasitol. 32, 1183–1191. (doi:10.1016/S0020-7519(02)00066-8) 12117501 10.1016/s0020-7519(02)00066-8

[30] Sebé-Pedrós A, Irimia M, del Campo J, Parra-Acero H, Russ C, Nusbaum C, Blencowe BJ, Ruiz-Trillo I 2013 Regulated aggregative multicellularity in a close unicellular relative of metazoa. Elife 2, e01287 (doi:10.7554/eLife.01287) 24368732 10.7554/eLife.01287 PMC3870316

[31] Altschul SF, Gish W, Miller W, Myers EW, Lipman DJ 1990 Basic local alignment search tool. J. Mol. Biol. 215, 403–410. (doi:10.1016/S0022-2836(05)80360-2) 2231712 10.1016/S0022-2836(05)80360-2

[32] Summons RE, Bradley AS, Jahnke LL, Waldbauer JR 2006 Steroids, triterpenoids and molecular oxygen. Phil. Trans. R. Soc. B 361, 951–968. (doi:10.1098/rstb.2006.1837) 16754609 10.1098/rstb.2006.1837 PMC1578733

[33] Zerenturk EJ, Sharpe LJ, Ikonen E, Brown AJ 2013 Desmosterol and DHCR24: unexpected new directions for a terminal step in cholesterol synthesis. Prog. Lipid Res. 52, 666–680. (doi:10.1016/j.plipres.2013.09.002) 24095826 10.1016/j.plipres.2013.09.002

[34] Najle SR, Nusblat AD, Nudel CB, Uttaro AD 2013 The sterol-C7 desaturase from the ciliate tetrahymena thermophila is a Rieske oxygenase, which is highly conserved in animals. Mol. Biol. Evol. 30, 1630–1643. (doi:10.1093/molbev/mst076) 23603937 10.1093/molbev/mst076

[35] Yoshiyama-Yanagawa T et al. 2011 The conserved Rieske oxygenase DAF-36/Neverland is a novel cholesterol-metabolizing enzyme. J. Biol. Chem. 286, 25 756–25 762. (doi:10.1074/jbc.M111.244384) 10.1074/jbc.M111.244384 PMC3138242 21632547

[36] Hanner M, Moebius FF, Flandorfer A, Knaus HG, Striessnig J, Kempner E, Glossmann H 1996 Purification, molecular cloning, and expression of the mammalian sigma1-binding site. Proc. Natl Acad. Sci. USA 93, 8072–8077. (doi:10.1073/pnas.93.15.8072) 8755605 10.1073/pnas.93.15.8072 PMC38877

[37] Rahier A, Pierre S, Riveill G, Karst F 2008 Identification of essential amino acid residues in a sterol 8,7-isomerase from *Zea mays* reveals functional homology and diversity with the isomerases of animal and fungal origin. Biochem. J. 414, 247–259. (doi:10.1042/BJ20080292) 18459942 10.1042/BJ20080292

[38] Moebius FF, Fitzky BU, Wietzorrek G, Haidekker A, Eder A, Glossmann H 2003 Cloning of an emopamil-binding protein (EBP)-like protein that lacks sterol Δ8-Δ7 isomerase activity. Biochem. J. 374, 229–237. (doi:10.1042/BJ20030465) 12760743 10.1042/BJ20030465 PMC1223579

[39] Chen W, Lee M-K, Jefcoate C, Kim S-C, Chen F, Yu J-H 2014 Fungal cytochrome p450 monooxygenases: their distribution, structure, functions, family expansion, and evolutionary origin. Genome Biol. Evol. 6, 1620–1634. (doi:10.1093/gbe/evu132) 24966179 10.1093/gbe/evu132 PMC4122930

[40] Dupont S, Lemetais G, Ferreira T, Cayot P, Gervais P, Beney L 2012 Ergosterol biosynthesis: a fungal pathway for life on land? Evolution 66, 2961–2968. (doi:10.1111/j.1558-5646.2012.01667.x) 22946816 10.1111/j.1558-5646.2012.01667.x

[41] Rottiers V, Motola DL, Gerisch B, Cummins CL, Nishiwaki K, Mangelsdorf DJ, Antebi A 2006 Hormonal control of *C. elegans* dauer formation and life span by a Rieske-like oxygenase. Dev. Cell 10, 473–482. (doi:10.1016/j.devcel.2006.02.008) 16563875 10.1016/j.devcel.2006.02.008

[42] Yoshiyama T, Namiki T, Mita K, Kataoka H, Niwa R 2006 Neverland is an evolutionally conserved Rieske-domain protein that is essential for ecdysone synthesis and insect growth. Development 133, 2565–2574. (doi:10.1242/dev.02428) 16763204 10.1242/dev.02428

[43] Xu X, London E 2000 The effect of sterol structure on membrane lipid domains reveals how cholesterol can induce lipid domain formation. Biochemistry 39, 843–849. (doi:10.1021/bi992543v) 10653627 10.1021/bi992543v

[44] Olson BJSC 2013 From brief encounters to lifelong unions. Elife 2, e01893 (doi:10.7554/eLife.01893) 24368736 10.7554/eLife.01893 PMC3870315

[45] Zweytick D, Hrastnik C, Kohlwein SD, Daum G 2000 Biochemical characterization and subcellular localization of the sterol C-24(28) reductase, Erg4p, from the yeast *Saccharomyces cerevisiae*. FEBS Lett. 470, 83–87. (doi:10.1016/S0014-5793(00)01290-4) 10722850 10.1016/s0014-5793(00)01290-4

[46] Nes WD 2003 Enzyme mechanisms for sterol C-methylations. Phytochemistry 64, 75–95. (doi:10.1016/S0031-9422(03)00349-2) 12946407 10.1016/s0031-9422(03)00349-2

[47] Zhou W, Lepesheva GI, Waterman MR, Nes WD 2006 Mechanistic analysis of a multiple product sterol methyltransferase implicated in ergosterol biosynthesis in *Trypanosoma brucei*. J. Biol. Chem. 281, 6290–6296. (doi:10.1074/jbc.M511749200) 16414960 10.1074/jbc.M511749200

[48] Nes WD, Xu S, Haddon WF 1989 Evidence for similarities and differences in the biosynthesis of fungal sterols. Steroids 53, 533–558. (doi:10.1016/0039-128X(89)90030-5) 2678609 10.1016/0039-128x(89)90030-5

[49] Chitwood DJ, Lozano R, Lusby WR 1986 Recent developments in nematode steroid biochemistry. J. Nematol. 18, 9–17. 19294131 PMC2618509

[50] Bergquist PR, Karuso P, Cambie RC, Smith DJ 1991 Sterol composition and classification of the Porifera. Biochem. Syst. Ecol. 19, 17–24. (doi:10.1016/0305-1978(91)90109-D)

[51] Roue M, Quevrain E, Domart-Coulon I, Bourguet-Kondracki M-L 2012 Assessing calcareous sponges and their associated bacteria for the discovery of new bioactive natural products. Nat. Prod. Rep. 29, 739–751. (doi:10.1039/c2np20040f) 22660834 10.1039/c2np20040f

[52] Shetty PH, Fried B, Sherma J 1990 Sterols in the plasma and digestive gland–gonad complex of *Biomphalaria glabrata* snails, fed lettuce versus hen's egg yolk, as determined by GLC. Comp. Biochem. Physiol. B. 96, 791–794. 2225776 10.1016/0305-0491(90)90233-j

[53] Haubrich BA et al. 2015 Discovery of an ergosterol-signaling factor that regulates *Trypanosoma brucei* growth. J. Lipid Res. 56, 331–341. (doi:10.1194/jlr.M054643) 25424002 10.1194/jlr.M054643 PMC4306687

[54] Nes CR, Singha UK, Liu J, Ganapathy K, Villalta F, Waterman MR, Lepesheva GI, Chaudhuri M, Nes WD 2012 Novel sterol metabolic network of *Trypanosoma brucei* procyclic and bloodstream forms. Biochem. J. 443, 267–277. (doi:10.1042/BJ20111849) 22176028 10.1042/BJ20111849 PMC3491665

[55] Conner RL, Landrey JR, Burns CH, Mallory FB 1968 Cholesterol inhibition of pentacyclic triterpenoid biosynthesis in *Tetrahymena pyriformis*. J. Protozool. 15, 600–605. (doi:10.1111/j.1550-7408.1968.tb02178.x) 5703082 10.1111/j.1550-7408.1968.tb02178.x

[56] Krauss K, Altevogt P 1999 Integrin leukocyte function-associated antigen-1-mediated cell binding can be activated by clustering of membrane rafts. J. Biol. Chem. 274, 36 921–36 927. (doi:10.1074/jbc.274.52.36921) 10601245 10.1074/jbc.274.52.36921

[57] Simons K, Toomre D 2000 Lipid rafts and signal transduction. Nat. Rev. Mol. Cell Biol. 1, 31–39. (doi:10.1038/35036052) 11413487 10.1038/35036052

[58] Trapnell C et al. 2012 Differential gene and transcript expression analysis of RNA-seq experiments with TopHat and Cufflinks. Nat. Protoc. 7, 562–578. (doi:10.1038/nprot.2012.016) 22383036 10.1038/nprot.2012.016 PMC3334321

[59] Anders S, Huber W 2010 Differential expression analysis for sequence count data. Genome Biol. 11, R106 (doi:10.1186/gb-2010-11-10-r106) 20979621 10.1186/gb-2010-11-10-r106 PMC3218662

[60] Robinson MD, McCarthy DJ, Smyth GK 2010 edgeR: a Bioconductor package for differential expression analysis of digital gene expression data. Bioinformatics 26, 139–140. (doi:10.1093/bioinformatics/btp616) 19910308 10.1093/bioinformatics/btp616 PMC2796818

[61] Tarazona S, Garcia-Alcalde F, Dopazo J, Ferrer A, Conesa A 2011 Differential expression in RNA-seq: a matter of depth. Genome Res. 21, 2213–2223. (doi:10.1101/gr.124321.111) 21903743 10.1101/gr.124321.111 PMC3227109

[62] Bligh EG, Dyer WJ 1959 A rapid method of total lipid extraction and purification. Can. J. Biochem. Physiol. 37, 911–917. (doi:10.1139/o59-099) 13671378 10.1139/o59-099

[63] Entezami AA, Venables BJ, Daugherty KE 1987 Analysis of lipids by one-dimensional thin-layer chromatography. J. Chromatogr. 387, 323–331. (doi:10.1016/S0021-9673(01)94535-2) 3558628 10.1016/s0021-9673(01)94535-2

