# Supplementary material for: Sterol metabolism in the filasterean Capsaspora owczarzaki has features that resemble both fungi and animals
Source: Open Biol. 2016 Jul 6;6(7):160029. doi: 10.1098/rsob.160029 (PMC4967820; doi:10.1098/rsob.160029)
Supplement: Supplementary Table, Figures and Figures Legends (S1–S4) [file rsob160029supp1.pdf]

Supplementary Table, Figures and Figure Legends (S1-S4)

Supplementary Table S1. Sterol composition of *C. owczarzaki* adherent and cystic cells obtained by GC-MS analyses (Relative %).

Compound [a]	**Adherent**	**Cystic**
**1**	0,34	0,49
**2**	0,09	3,22
**3**	5,04	4,67
**4**	84,17	75,01
**5**	2,04	2,84
**6**	0,15	0,44
**7**	7,76	13,05
**8**	0,08	0,05
**9**	0,14	0,09
**10**	0,19	0,14

[a] Sterol identity numbers are the same as in Table 2.

Supplementary Figure S1.

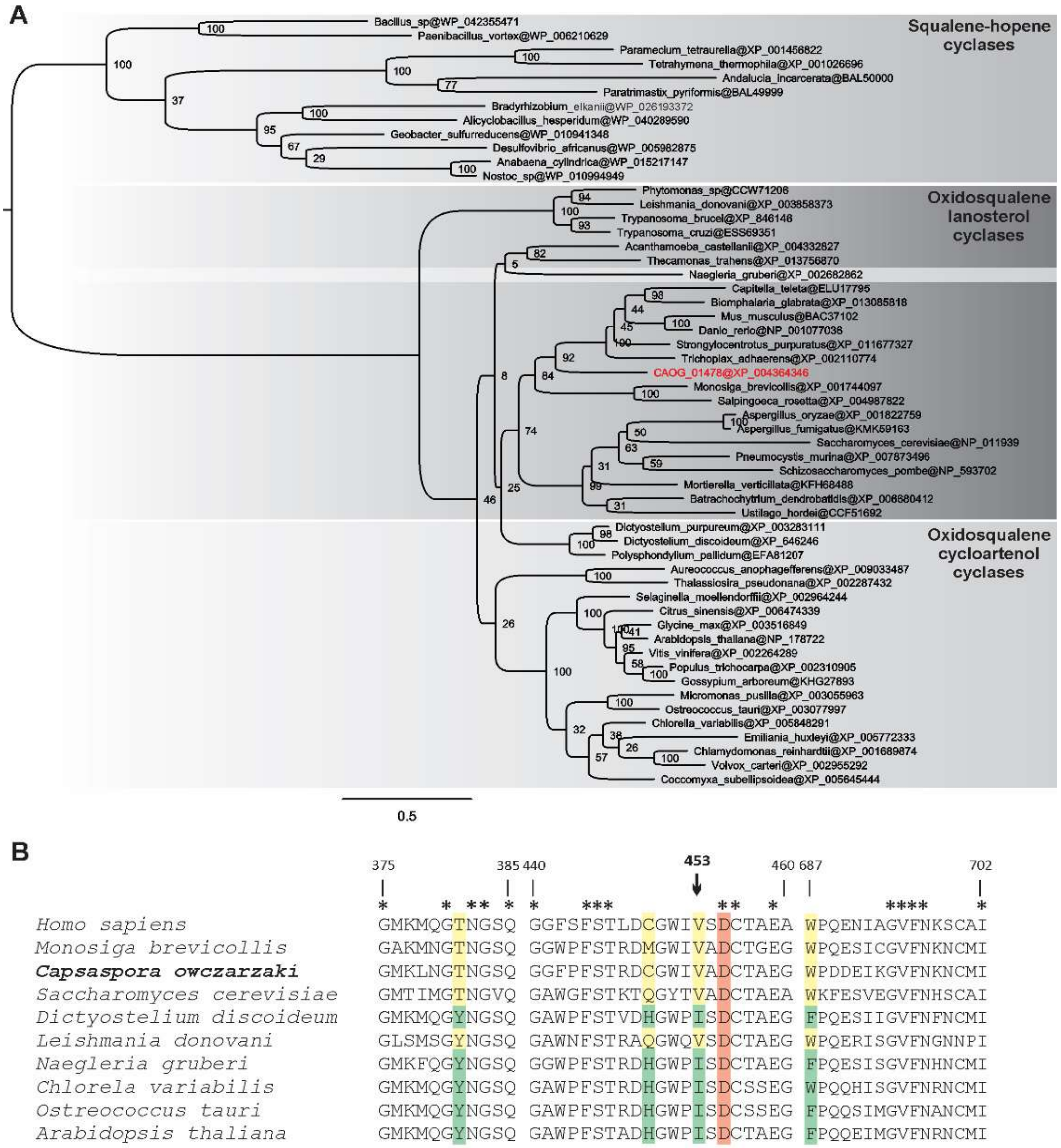

Supplementary Figure S2.

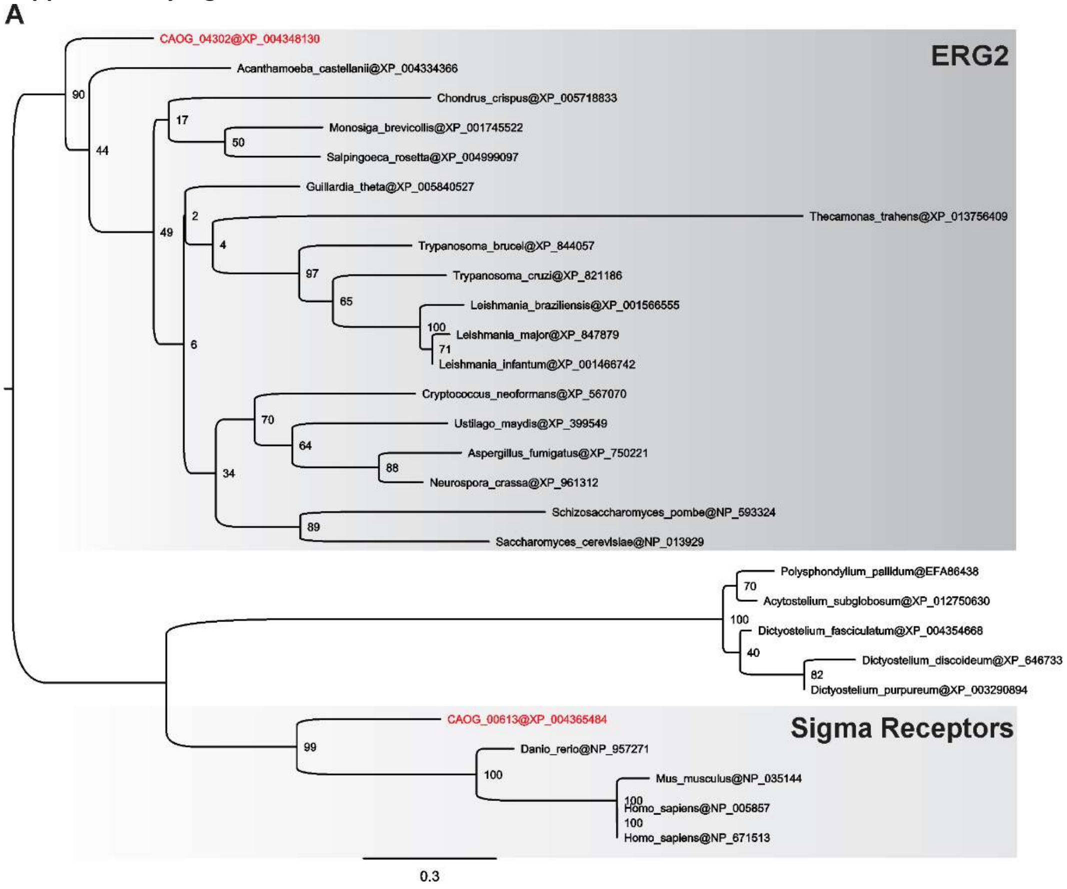

B

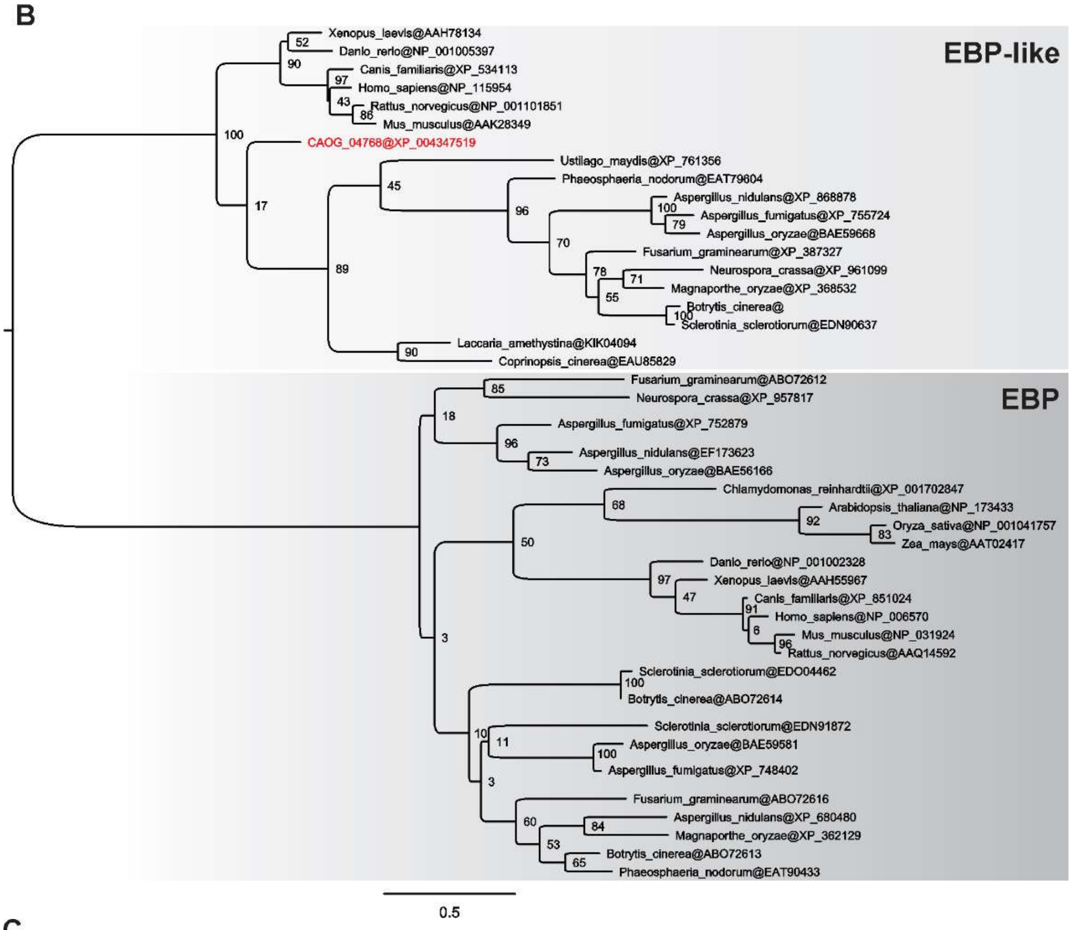
EBP-like
Xenopus_laevis@AAH78134
Danio_rerio@NP_001005397
Canis_familiaris@XP_534113
Homo_sapiens@NP_115954
Rattus_norvegicus@NP_001101851
Mus_musculus@AAK28349
CAOG_04768@XP_004347519
Ustilago_maydis@XP_761356
Phaeosphaeria_nodorum@EAT79604
Aspergillus_nidulans@XP_868878
Aspergillus_fumigatus@XP_755724
Aspergillus_oryzae@BAE59668
Fusarium_graminearum@XP_387327
Neurospora_crassa@XP_961099
Magnaporthe_oryzae@XP_368532
Botrytis_cinerea@
Sclerotinia_sclerotiorum@EDN90637
Laccaria_amethystina@KIK04094
Coprinopsis_cinerea@EAU85829
EBP
Fusarium_graminearum@ABO72612
Neurospora_crassa@XP_957817
Aspergillus_fumigatus@XP_752879
Aspergillus_nidulans@EF173623
Aspergillus_oryzae@BAE56166
Chlamydomonas_reinhardtii@XP_001702847
Arabidopsis_thaliana@NP_173433
Oryza_sativa@NP_001041757
Zea_mays@AAT02417
Danio_rerio@NP_001002328
Xenopus_laevis@AAH55967
Canis_familiaris@XP_851024
Homo_sapiens@NP_006570
Mus_musculus@NP_031924
Rattus_norvegicus@AAQ14592
Sclerotinia_sclerotiorum@EDO04462
Botrytis_cinerea@ABO72614
Sclerotinia_sclerotiorum@EDN91872
Aspergillus_oryzae@BAE59581
Aspergillus_fumigatus@XP_748402
Fusarium_graminearum@ABO72616
Aspergillus_nidulans@XP_680480
Magnaporthe_oryzae@XP_362129
Botrytis_cinerea@ABO72613
Phaeosphaeria_nodorum@EAT90433
0.5

C

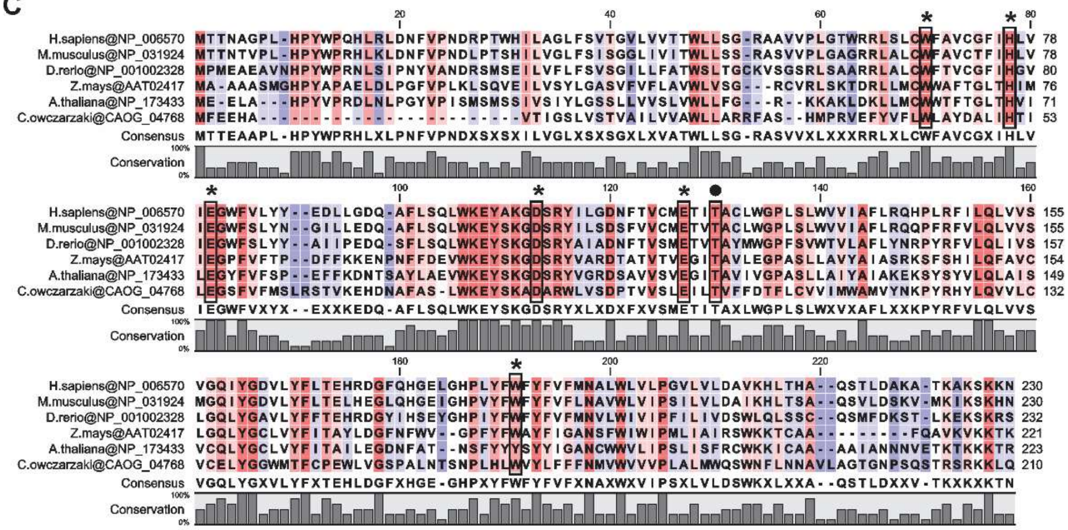
H.sapiens@NP_006570
M.musculus@NP_031924
D.rerio@NP_001002328
Z.mays@AAT02417
A.thaliana@NP_173433
C.owczarzaki@CAOG_04768
Consensus
Conservation

Supplementary Figure S3.

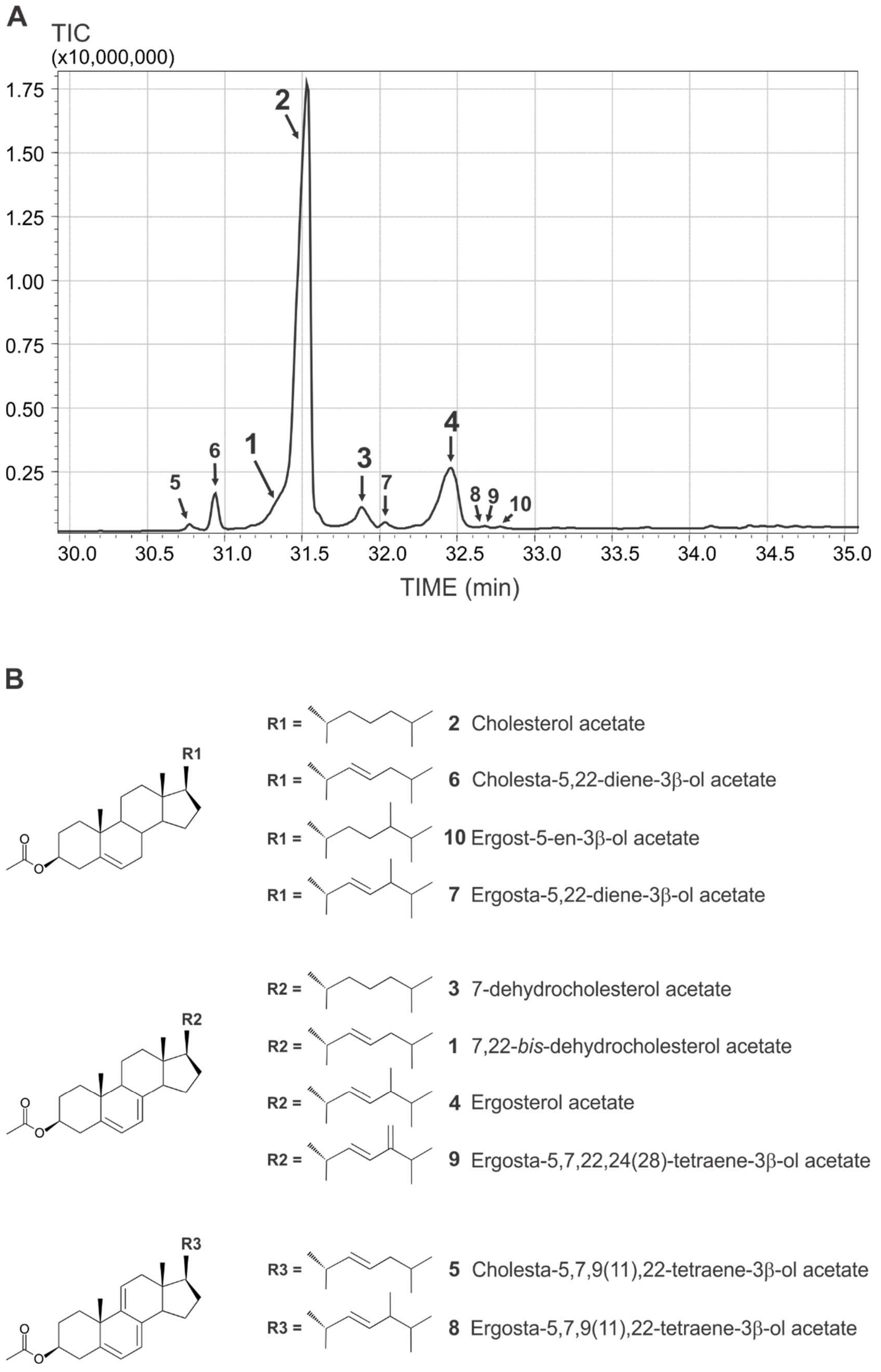

Supplementary Figure S4.

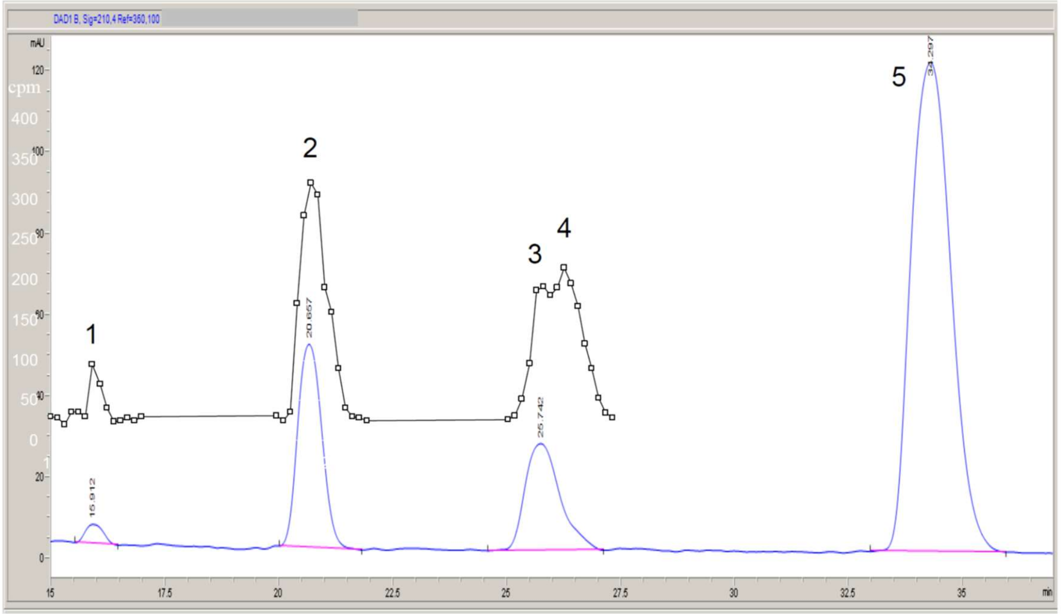

Supplementary Figure Legends (S1-S4)

Fig. S1. A. ML analysis of oxidosqualene cyclases. Bootstrap supports are indicated. *C. owczarzaki* OSC is highlighted in red. Protein IDs are indicated in the branch labels. **B.** Partial alignment of OSCs. Numbering corresponds to positions in the *Homo sapiens* protein. The catalytic residue D is highlighted in red. Asterisks denote conserved amino acids. Residues in positions 389, 441, 453 and 687 are differentially conserved between oxidosqualene-lanosterol cyclases (yellow) and oxidosqualene-cycloartenol cyclases (green).

Fig. S2. A-B. Phylogenetic analysis of sterol Δ8,Δ7-isomerases. **A.** ML tree of ERG2-Sigma 1 family of proteins. **B.** ML analysis of Emopamil Binding Protein family. *C. owczarzaki* homologs are indicated in red. **C.** Multiple sequence alignment of EBP proteins. Residues are colored according to their percentage of conservation. Asterisks denote essential amino acid residues, according to (Rahier et al. 2008).

Fig. S3. A. Total Ion Chromatogram (TIC) of acetylated sterols obtained from *C. owczarzaki* cells grown for 7 days in ATCC 1034 medium (cystic cells). **B.** Structures of all the sterols species identified in the GC/MS analysis. Numbers in bold correspond to the respective peaks indicated in Figs. 4A and S3A.

Fig. S4. HPLC analysis of sterols from *C. owczarzaki* filopodial stage. Cells were cultured for 4 days in ATCC 1034 medium with the addition of ^{14}C-cholesterol, as described in Materials and Methods. Blue line indicates absorbance at 210 nm. Black line corresponds to radioactivity (cpm) and it is shown from 15 to 27 min to simplify the figure. Peak 1 showed significant absorbance also at 325 nm, suggesting it could represent a conjugated triply unsaturated sterol, no further characterized. Peaks 2 to 4 significantly absorbed at 280 nm, suggestive of conjugated doubly unsaturated sterols. Their retention times corresponded, respectively, to those of standards of 7,22-*bis*-dehydrocholesterol (20.6 min), ergosterol (25.6 min), and 7-dehydrocholesterol (26.1 min). Peak 5 eluted, like cholesterol standard, at 34.3 min.

References

Rahier, A., Pierre, S., Riveill, G. & Karst, F. 2008 Identification of essential amino acid residues in a sterol 8,7-isomerase from Zea mays reveals functional homology and diversity with the isomerases of animal and fungal origin. *Biochem. J.* **414**, 247–259. (doi:10.1042/BJ20080292)
